# Does the pre-operative buccal soft tissue level at teeth or gingival phenotype dictate the aesthetic outcome after flapless immediate implant placement and provisionalization? Analysis of a prospective clinical case series

**DOI:** 10.1186/s40729-021-00366-3

**Published:** 2021-08-27

**Authors:** Edith Groenendijk, Ewald Maria Bronkhorst, Gert Jacobus Meijer

**Affiliations:** 1 Clinique Implantologie Den Haag, Stadhouderslaan 12, 2517 HW Den Haag, The Netherlands; 2 Department of Preventive and Curative Dentistry, Radboudumc, Nijmegen, The Netherlands; 3 Department of Oral and Maxillofacial Surgery, Radboudumc, Nijmegen, The Netherlands

**Keywords:** Immediate implant placement and provisionalization, Immediate restoration, Aesthetic outcome, Modified pink esthetic score, Soft tissue level, Mid-buccal recession, Pink aesthetic score, Flapless implant surgery, IIP, IIPP

## Abstract

**Background:**

Immediate implant placement (IIP) often is related to mid-buccal recession in literature. To draw conclusions about the behavior of the soft tissues following IIP, pre-operative aesthetic measurements have to be taken into account. The aim of analysis of these prospective clinical case series data was to elucidate whether the pre-operative buccal soft tissue level (STL) or gingival phenotype influence the 1-year pink aesthetic outcome after performing flapless immediate implant placement and provisionalization (FIIPP) maxillary incisor cases.

**Materials and methods:**

In 97 patients, a maxillary incisor was replaced performing FIIPP. STL and phenotype were analyzed on light-photographs made pre-operatively (T^0^), direct post-operatively (T^1^), after placement of the permanent crown (T^2^), and 1 year post-operatively (T^3^). To investigate if a pre-operative buccal soft tissue deficiency or excess influenced the total pink esthetic score (total-PES) per patient at T^3^, PES-3 was modified by adding a minus (“−”) or plus (“+”) in case of a STL-deficiency or excess, respectively.

**Results:**

Pre-operatively, 40% of the cases showed a mid-buccal recession (STL-deficiency), 19% STL-excess, while in 41% an equal level in comparison with the contra-lateral tooth was observed (STL-neutral). One year post-operatively, 79% (31/39) of the recession cases showed soft tissue gain, while STL-excess cases showed the highest rate of soft tissue reduction (94%; 17/18). This resulted in a decrease of soft tissue recessions and excesses (to 26% and 4%, respectively), and an increase of ideal STL (PES-3-score 2) to 70%. The 1-year aesthetic outcome was not statistically different (p = 0.577) between patients with a pre-operative soft tissue recession (mean T^3^ total-PES = 12.18) or STL excess (mean T^3^ total-PES = 11.94). Of the total population, 71 patients with a thin, and 26 with a thick phenotype were evaluated. No statistical difference (p = 0.08) was present in aesthetic outcome between the two phenotypes (thin mean T^3^ total-PES = 12.30, thick mean T^3^ total-PES = 11.65).

**Conclusion:**

Regardless of phenotype, preoperative soft tissue recession, or excess, comparable high aesthetic outcomes were achieved 1 year post-operatively.

**Trial registration:**

Ethical approval was obtained and registered on 20 October 2015 (NTR5583/NL4170).

## Background

The risk of a poor pink aesthetic outcome of immediately placed implants often is related to mid-buccal recession in literature [1–3]. Surgical and restorative approaches, as also implant position, are pivotal in achieving an optimal aesthetic outcome, particularly on the point of the mid-buccal soft tissue level.

Hardly any of the studies on the field of the aesthetic outcome after implant therapy are comparable with each other considering heterogeneity of treatment protocols, implant position, materials, and aesthetic scores used. To evaluate the soft tissues around dental implants, in 2005, the Pink Esthetic Score (PES) was introduced by Fürhauser et al. [4]; soft tissues are judged using seven criteria which each can be scored by 0, 1, or 2. Therefore, the total pink aesthetic score (total-PES) ranges from 0 to 14 (total-PES_1–14_). This method is applied in many researches [5–15] and generally preferred over the simplified-PES, introduced by Belser et al. [16]. This latter was launched in favor of ‘ease of use and understanding’ by varying the total-PES score between 1 and 10 (total-PES_1–10_). For this goal, important information is sacrificed by merging the original criteria “alveolar deficiency,” “soft tissue color,” and “soft tissue texture.”

To draw conclusions about the behavior of the soft tissues following IIP, pre-operative aesthetic measurements have to be taken into account. In our prospective cases series, we reported a high over-all aesthetic outcome (total-PES = 12.1) 1 year after FIIPP [15]. This analysis elucidates whether the pre-operative buccal soft tissue level (STL) or gingival phenotype influences the 1-year pink aesthetic outcome after performing FIIPP in single tooth maxillary incisor cases.

## Material and methods

In a prospective clinical case series, 100 consecutive patients were treated with flapless immediate implant placement and provisionalization (FIIPP), due to a failing maxillary incisor. The CARE reporting guidelines were used [17]. Inclusion criteria were (1) presence of one failing single maxillary incisor in between two neighboring healthy teeth, (2) sufficient occlusal support, (3) absence of periodontal disease, (4) absence of bruxism, (5) existence of an adequate bone height at the apical area of the socket (at least 5 mm) to allow primary implant stability. Intact sockets, as also sockets with a peri-apical bone defect or a crestal bone defect not exceeding 5 mm, were included. Reasons for extraction comprised crown or root fracture, root resorption, caries, and persisting endodontic pathology. Exclusion criteria were (1) smoking habits exceeding more than 10 units a day, (2) pregnancy, (3) bone diseases or a history of irradiation, (4) ASA III or higher. Both surgical and restorative procedures were performed following a standardized protocol [13, 15].

### Pink aesthetic outcome and gingival phenotype

Both the implant and contra-lateral site were photographed in a standardized way [18] at different time points; pre-operatively (T^0^), 7–14 days post-operatively (T^1^), direct after placement of the permanent crown (T^2^), and 1 year post-operatively (T^3^). On each time point, two light photographs were taken: one perpendicular to the mid-buccal of the tooth arch, and one perpendicular to the implant site. Before examination, the light photographs were placed in a digital format. Evaluation of the pink aesthetic outcome was executed as described by Fürhauser et al. [4], by two blinded examiners, who were not involved in the patient treatments. The same was true for the phenotype analysis. The inter-examiner reliability showed an intra-class correlation coefficient (ICC) of 0.979 for the PES.

### STL measurements

For these measurements, light photographs perpendicular to the tooth arch at T^0^, T^1^, T^2^, and T^3^ were used and placed into a digital format. Reference lines were drawn through, gingival margin of the contra-lateral incisor, incisal edge of contra-lateral incisor, and distal from the central and lateral incisors. The gingival margin of the failing tooth at T^0^ was drawn in blue as a reference at different time points.

In order to enlighten if the pre-operative buccal STL influenced the total-PES per patient at T^3^, the PES-3 of the total pink aesthetic score (PES) [4] was modified. The original PES-3 index by Fürhauser et al. (2005) only describes a discrepancy in the STL. Whether this is positive (excess) or negative (deficiency) remains unclear. For instance, a site can show an excess of soft tissue of 1–2 mm pre-operatively and show a deficiency of 1–2 mm 1 year post-operatively; however, in both situations, the PES-3 score is 1. Our proposal is to change the PES-3 into the modified PES-3 (mPES-3); a minus (“−”) is added to the score when a STL deficiency is observed, and a plus (“+”) if an excess of soft tissue is present. The exact method is presented in Table 1. In case of STL-deficiency, a “minus” sign to PES-3-score was added, and in case of a STL-excess, a “plus” sign. As reference, always the contra-lateral reference tooth was used.

**Table 1:** Modified PES-3 (mPES-3); in case of a deficiency, a minus sign, and in case of a surplus, a plus sign is added behind the original PES-3 score

Modified PES-3 (mPES-3): level of soft-tissue margin versus reference tooth				
0−	**1**−	**2**	**1+**	**0+**
Major deficiency > 2 mm	**Minor deficiency** **1–2 mm**	**No discrepancy** **> 1 mm**	**Minor surplus** **1–2 mm**	**Major surplus** **> 2 mm**

### Statistical methods

Total-PES at different time points T^0^, T^1^, T^2^, and T^3^ of patients with a pre-operative soft tissue recession or excess, as well as biotype were compared and tested on significant difference using Levene’s test for equality of variances and t test for equality of means. Statistical significance was defined as p = 0.05.

## Results

Of the 100 included patients, 97 were available for evaluation; 1 was excluded because the implant site was traumatized; the already installed implant was replaced by a new one. Another patient withdrew because of relocation. Of the third patient, the light photos were missing at T^0^. The remaining 97 patients consisted of 56 females and 41 males with a mean age of 46 years (range 17–80 years). Unfortunately, eight light photos were missing at T^1^, and seven at T^2^.

### STL measurements

**Fig. 1:**
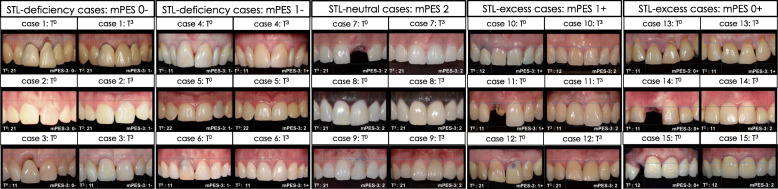
Three examples of cases with comparable pre-operative soft tissue level (STL) and their STL at T^3^; STL-deficiency > 2 mm (mPES = 0−), STL-deficiency 1–2 mm (mPES = 1−), STL-neutral (mPES = 2), STL-excess (mPES = 1+), and STL-excess (mPES = 0+)

**Fig. 2:**
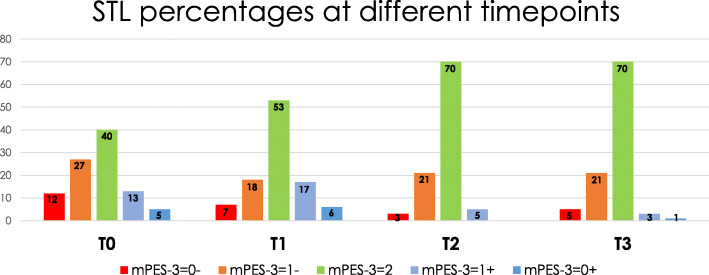
Distribution of cases per STL-group (mPES-3 = 0−, 1−, 2, 1+, 0+) per time point in percentages. Red (mPES = 0−) and orange (mPES = 1−) concern STL-deficiency, green (mPES = 2) is optimal STL-neutral, light (mPES = 1+) and dark blue (mPES = 0+) concern STL-excess

Examples of 3 cases per pre-operative STL and their modified PES-3 scores (mPES-3) at T^0^/T^3^ are shown in Fig. 1. Distribution of cases per STL-group (mPES-3 = 0−, 1−, 2, 1+, 0+) per timepoint is shown in Fig. 2.

Pre-operatively, 40% of the cases showed a mid-buccal recession (STL-deficiency), 19% STL-excess, while in 41% an equal level in comparison with the contra-lateral tooth was observed (STL-neutral). One year post-operatively, 79% (31/39) of the recession cases showed soft tissue gain, while STL-excess cases showed the highest rate of soft tissue reduction (94%; 17/18). This resulted in a decrease of soft tissue recessions and excesses (to 26% and 4%, respectively), and an increase of ideal STL (PES-3 = 2) to 70%.

Comparing 1-year pink aesthetic outcome of cases with a baseline STL-deficiency (mean T^3^ total-PES = 12.18) or STL-excess (mean T^3^ total-PES = 11.94 ), there was no statistical difference (p = 0.577) found (Table 2). Thus, whether a pre-operative STL-deficiency or STL-excess was present, it did not affect the pink aesthetic outcome 1 year post-operatively.

**Table 2:** Comparison of mean total-PES for all points in time, based on surplus, or deficiency of STL at T^0^. Equal variances were assumed

Group statistics					T test for equality of means			
		N	Mean	SD	p value (2-tailed)	Mean difference	95% Confidence interval of the difference	
							Lower	Upper
Total-PES T^0^	**STL deficiency at T**^**0**^	39	**9.23**	2.64	0.946	− 0.05	− 1.44	1.35
	**STL excess at T**^**0**^	18	**9.28**	1.93				
Total-PES T^1^	**STL deficiency at T**^**0**^	35	**10.54**	2.36	0.442	0.48	− 0.77	1.74
	**STL excess at T**^**0**^	17	**10.06**	1.48				
Total-PES T^2^	**STL deficiency at T**^**0**^	37	**11.62**	1.38	0.883	− 0.07	− 0.96	0.83
	**STL excess at T**^**0**^	16	**11.69**	1.70				
Total-PES T^3^	**STL deficiency at T**^**0**^	39	**12.18**	1.49	0.577	0.24	− 0.60	1.07
	**STL excess at T**^**0**^	18	**11.94**	1.43				

### Gingival phenotype

Of the total population, 71 patients with a thin phenotype and 26 with a thick phenotype were found (Table 3). There was no statistical difference (p = 0.079) in aesthetic outcome found between patients with a thin (mean T^3^ total-PES = 12.30) or thick phenotype (mean T^3^ total-PES = 11.65).

**Table 3:** Effect of biotype on pink aesthetic outcome 1 year post-operatively

Group statistics					T test for equality of means			
	Biotype	N	Mean	SE mean	p value (2-tailed)	Mean difference	95% Confidence interval of the difference	
							Lower	Upper
Total-PES T^3^	**0 = thin**	71	**12.30**	0.162	0.079	0.642	− 0.076	1.36
	**1 = thick**	26	**11.65**	0.404				

## Discussion

A difference in pink aesthetic outcome was expected between cases showing a pre-operative STL-excess, a pre-operative STL-neutral, or pre-operative STL-deficiency. Especially for the latter, a lower total-PES was expected. However, the contrary appeared true; after performing FIIPP, also for the STL-deficiency cases a not significant different high total-PES score was noted. As such, the pre-operative STL at teeth did not influence the overall pink aesthetic outcome 1 year post-operatively.

In cases with a pre-operative excess of soft tissue comparing to the contra-lateral incisor, reduction of soft tissue height is required. In 15 cases, the desired STL reduction was reached to level up with the contra-lateral tooth. This was obtained by less filling of the socket with bone substitute and/or by lateral pressure of the abutment/crown onto the soft tissue during placement. It has to be taken into account that the soft tissues after socket-grafting by application of a bone substitute are less resilient. That is probably the reason that in one, case the surplus of soft tissue remained. In the two recession cases of the “excess” group (from mPES-3 T^0^ = 1+ to mPES-3 T^3^ = 1−), probably too much lateral pressure of the supra-structure onto the tissues caused a undesired soft tissue deficiency. Despite of this, in 100% of the cases, the same or better PES-3 score was achieved. Appliance of the mPES-3 clarified what really happened with the STL.

Cases starting with a STL-neutral seemed to be most challenging to treat. In this group, the pre-operative STL is already optimal, the soft tissues have only to be preserved, however, not over- or under-contoured. A slight change of the soft tissue will result in a lesser aesthetic outcome. The post-operative recessions probably are a result of bone-substitute leakage due to post-operative bleeding, or placement of a too bulky permanent abutment causing pressure onto the surrounding hard- and soft tissues.

Surprisingly, STL gain occurred in cases were pre-operatively a STL-deficiency (recession) was present. This is in confirmation with the findings of Noelken et al. [19]. They performed immediate implant placement and provisionalization (IIPP) on a single maxillary tooth with a pre-operative recession in 26 patients, of which 13 were treated with a connective tissue graft, and 13 without such graft. After a mean follow-up period of 45 months, recessions were significantly reduced in both groups. In another study, in which IIPP in intact sockets and defect sockets was compared, similar data on the field of total-PES score were presented [20]. In both intact extraction sockets, as well as in alveoli with buccal bone defects, IIPP rendered similar outcomes with regard to total-PES, height of the buccal gingival margin, and peri-implant bone level after 1 year. Although their treatment protocol is different, these authors corroborate that recession sites can improve after IIPP. Multiple other authors already stressed that, due to performing ridge preservation, a gain in alveolar height was observed [21, 22]. This implicates that in our study, ridge preservation was not disturbed by immediate implant placement.

It is unclear how our results align with other IIPP or early and delayed placement protocol studies. In 2015, Schropp and Isodor [23] published that 1 to 1.5 years after performing early or delayed implant placement, less than 60% of the cases showed an appropriate crown length, thus STL-level. In addition, they stated that early placed implants tended to be superior to delayed-placed implants with respect to STL level. So cautiously, it can be stated that performing FIIPP conform this standardized protocol [13, 15] and shows better results at the point of soft tissue level than early or delayed protocols. Further research is necessary to confirm or decline these assumptions.

A thin gingival phenotype did not affect the pink aesthetic outcome. An explanation may be that by implants’ palatal positioning, a thick hard tissue crest is created.

A shortcoming of this study was that the STL was not measured in millimeter, but that a classification was used to monitor the STL, such as the PES. Unexpected was the high number of cases with a pre-operative STL-deficiency; indeed, it was possible to gain soft tissue without applying a soft tissue graft. Within the limitations of this prospective case series, we may conclude that patients with a small pre-operative STL-deficiency or STL-excess showed the same high aesthetic outcome (total-PES) as compared to cases with a pre-operative STL-neutral, 1-year post-operatively. Pre-operative mid-buccal recession, as well as STL-excess cases, tended to improve, while the cases with a neutral pre-operative STL were the most difficult to obtain.

## Data Availability

The datasets used and/or analyzed during the current study are available from the corresponding author on reasonable request.

## Abbreviations

IIP: Immediate implant placement
STL: Soft tissue level
FIIPP: Flapless immediate implant placement and provisionalization
PES: Pink aesthetic score
mPES: Modified pink aesthetic score
